# Biogeographical Survey Identifies Consistent Alternative Physiological Optima and a Minor Role for Environmental Drivers in Maintaining a Polymorphism

**DOI:** 10.1371/journal.pone.0032648

**Published:** 2012-02-27

**Authors:** Arne Iserbyt, Hans Van Gossum, Robby Stoks

**Affiliations:** 1 Evolutionary Ecology Group, Antwerp University, Antwerp, Belgium; 2 Laboratory of Aquatic Ecology and Evolutionary Biology, University of Leuven, Leuven, Belgium; Lund University, Sweden

## Abstract

The contribution of adaptive mechanisms in maintaining genetic polymorphisms is still debated in many systems. To understand the contribution of selective factors in maintaining polymorphism, we investigated large-scale (>1000 km) geographic variation in morph frequencies and fitness-related physiological traits in the damselfly *Nehalennia irene*. As fitness-related physiological traits, we investigated investment in immune function (phenoloxidase activity), energy storage and fecundity (abdomen protein and lipid content), and flight muscles (thorax protein content). In the first part of the study, our aim was to identify selective agents maintaining the large-scale spatial variation in morph frequencies. Morph frequencies varied considerably among populations, but, in contrast to expectation, in a geographically unstructured way. Furthermore, frequencies co-varied only weakly with the numerous investigated ecological parameters. This suggests that spatial frequency patterns are driven by stochastic processes, or alternatively, are consequence of highly variable and currently unidentified ecological conditions. In line with this, the investigated ecological parameters did not affect the fitness-related physiological traits differently in both morphs. In the second part of the study, we aimed at identifying trade-offs between fitness-related physiological traits that may contribute to the local maintenance of both colour morphs by defining alternative phenotypic optima, and test the spatial consistency of such trade-off patterns. The female morph with higher levels of phenoloxidase activity had a lower thorax protein content, and vice versa, suggesting a trade-off between investments in immune function and in flight muscles. This physiological trade-off was consistent across the geographical scale studied and supports widespread correlational selection, possibly driven by male harassment, favouring alternative trait combinations in both female morphs.

## Introduction

Polymorphisms are attractive model systems for understanding fundamental processes related to the origin and maintenance of genetic variation [1], [2]. For understanding processes maintaining polymorphisms, there is increasing awareness that, rather than focussing on one single mechanism, a combination of several adaptive and/or neutral mechanisms may determine polymorphism [3]–[7]. An ongoing debate in this context is the relative importance of neutral versus adaptive mechanisms [8]. One possibility to explore the relative contribution of these mechanisms is to compare genetic variability of neutral loci with geographic and temporal variation in morph frequencies [9]–[11]. Alternatively, one may evaluate to what extent spatiotemporal variation in morph frequencies and morph fitness correlates can be explained by variation in ecological parameters [12], [13].

Many polymorphisms show large-scale geographic variation in morph frequencies [14]–[18], which provides an elegant setting to explore co-varying ecological variables as indicators of spatially varying selection. For example, in the polymorphic snail *Littorina obtusata*, shell colour morph frequencies changed gradually in accordance with environmental temperature regimes within and between estuaries [13], suggesting that temperature acts as a major selective agent to maintain this colour polymorphism. Other examples in which morph frequencies relate to ecological variables involve niche occupancy in the barn owl, *Tito alba* [19], altitude related solar radiation in the polymorphic damselfly *Megalagrion calliphya* [20] and soil coloration to improve crypsis in *Agouti* mice [21]. In many cases, spatial morph frequency variation resembles a cline. However, such clines may also result from neutral processes [22]–[24]. To firmly point at spatial varying selection in maintaining polymorphisms, fitness-related traits should co-vary with the ecological variables in opposite ways between morphs, with a certain morph having optimal fitness-related traits at sites where it has the highest morph frequencies. For example, colour morphs of the walking stick *Timema cristinae* show spatial morph frequency variation related to the presence of host plants and the survival of a given morph is highest at the host plant where its frequency is highest [25].

While spatially varying selection may contribute to the maintenance of different morphs at large spatial scales, alternative fitness optima linked to trade-offs between fitness-related traits may underlie local coexistence of different morphs [26]. Fitness-related physiological traits that received special attention to explain the maintenance of polymorphisms are those related to immune function [27]–[30] and energy storage [31], [32]. Morphs may differentially trade off investments in fitness-related physiological traits, thereby generating alternative fitness optima in an adaptive landscape, caused by correlational selection on a multivariate suit of physiological and life-history traits [29], [30], [33], [34].

A classic and much debated example of intra-specific polymorphism can be found in many species of damselflies [35], [36]. In these systems, polymorphism is restricted to the female sex and shows simple Mendelian inheritance [37]. The observed biogeographic variation in female morph frequencies and the mechanisms underpinning these frequency dynamics remain an intriguing and controversial topic, e.g. [7], [15], [20], [38], [39]. To understand spatiotemporal patterns in female morph frequencies, partial support has been found for fluctuating selection pressure caused by male-female interactions [40], [41]. The presence of such distinct phenotypes are therefore generally explained in the context of sexual conflict theory, in which multiple female morphs co-exist as counter-adaptations to avoid costly male sexual harassment [42]–[44]. However in addition to this harassment-reduction hypothesis, recent studies point at a potential role for stochastic effects during recolonisation when it comes to explain morph frequency variation [45], differential dispersal capacity between female morphs [46], and especially differential preference or tolerance to local abiotic conditions [15], [20], [32], [47]. In support of the latter, a recent large scale population genetic study indicated the importance of divergent selection [7], in which certain morphs may be favoured in local populations that differ in ecological (biotic and abiotic) conditions. Specifically, the authors suggested potentially important factors like temperature and precipitation regimes that are likely to affect the different colour morphs in contrasting ways; see also [15], [32], [47]. Indeed, given the difference in body pigmentation, melanin pattern and behaviour it is not surprising that female morphs in these damselflies are expected to differ in thermal properties [32], [48]. Further supporting evidence comes from recent studies that showed significant clinal variation in female morph frequencies [15], [20], [29], [47], including co-variations with ambient temperature. Together, after several decades focusing on the harassment-reduction hypothesis, it became clear that one single hypothesis may not suffice to explain this polymorphism [5], [7].

In the current study, we used the polymorphic damselfly *Nehalennia irene*, a species with large geographical variation in morph frequencies [38], [49]. The stronger differentiation in morph frequencies at two sites, separated by only 8 km in Eastern Canada compared to genetic differentiation in neutral microsatellite markers indicated that divergent selection rather than neutral processes caused spatial variation in morph frequencies [50]. Yet, using this method one cannot identify which selective agents caused the geographic variation. Furthermore, morph frequencies appear to resemble a cline at continental scale from Northwest to Southeast Canada [49], which has been suggested to co-vary with both, ambient temperature and male density [38]. However, this suggested cline in based on groups of study populations with a discontinuity of sometimes more than 3000 km. Here, we elaborate on several previous studies [38], [45], [49], [50] and aim to identify selective agent(s) in maintaining this polymorphism. Therefore, we surveyed 89 populations along a linear 1100 km transect, representing a continuous ecological cline in population density, temperature and precipitation regimes. Following the St.-Laurence river in Eastern Canada provided us with an excellent opportunity to sample with consistent continuity.

In a first part of the study, our aim is to identify selective agents maintaining the large spatial variation in morph frequencies. If a female morph relative to the other, is favoured in local populations that differ in ecological parameters, we then expect (1) morph frequencies to co-vary with these ecological parameters [13], [19]–[21], [47], and (2) that in populations where this morph is favoured, it has more optimal values of fitness-related physiological traits, relative to the other morph [25], [32], [51]. The studied physiological traits involve key parameters related to investments in immune function, in energy storage and fecundity, and in flight muscles that are strong candidates to affect fitness in damselflies [52]–[55]. In a second, related part of the study, we aim at identifying trade-offs between fitness-related physiological traits that may contribute to the local maintenance of both colour morphs and test the spatial consistency of morph differences that define alternative phenotypic optima [29], [30], [33], [34].

## Materials and Methods

### Model species

The sedge sprite *N. irene* is a small non-territorial damselfly (Zygoptera; Odonata), which inhabits marshy or boggy waters and is common throughout most of Canada and the Northern parts of the United States [56]. It is not an endangered nor a protected species (see COSEWIC, federal government Canada). *Nehalennia irene* has one generation per year, with the winged adult life stage and reproduction typically occurring between early June and mid-August. After locating a potential mate, a male will attempt to grasp the female in the so-called tandem formation, where the male attaches his anal appendages to the female's prothorax [57]. In a next step, the male will then try to copulate with the female. A female's mate status can thus either be mated (tandem or copulating position) or either be single. The adult stage exhibits a clear dimorphism restricted to the female sex, with morphs being easily classified based on their body colouration and melanin pattern into andromorphs and gynomorphs [58]. Mature andromorph females resemble the conspecific male's blue body colouration and melanin pattern [59], [60], whereas gynomorph females have distinctive yellowish lateral thorax sides and a less conspicuous abdominal melanin pattern; for colour figures see [58], for pictures see [61]. Earlier research indicated large spatial but temporally fairly consistent variation in relative female morph frequencies, with proportions of andromorphs among females being atypically high at the Western edge of the species distribution range (>90%) relative to the central and Eastern part of the range (0–63%) [38], [49].

### Study sites and sampling procedure

**Figure 1:**
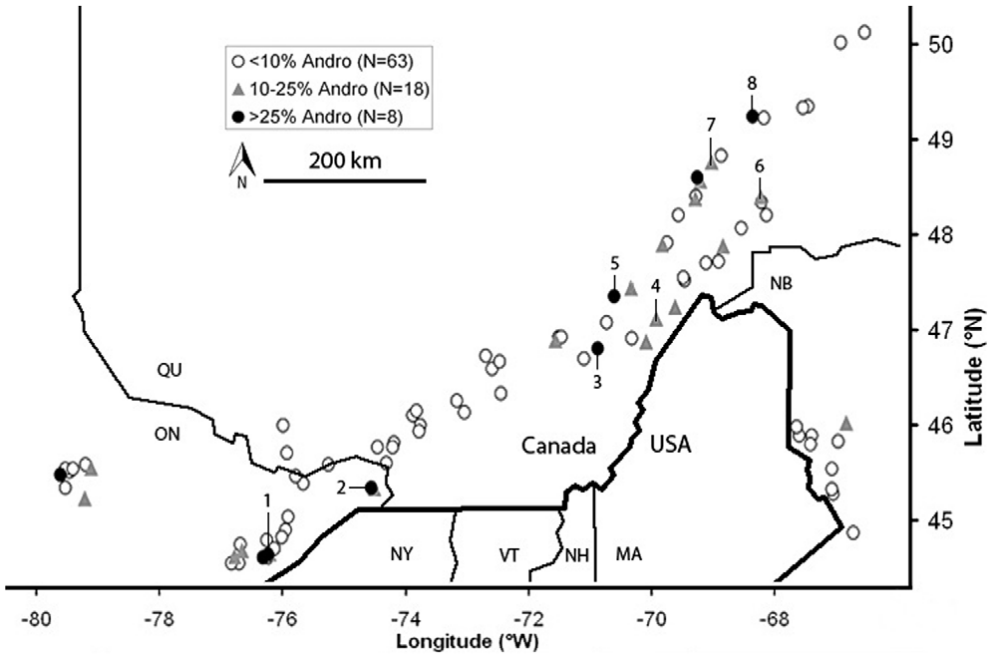
Geographical distribution of the study populations. The 89 populations are presented in three groups according to andromorph frequencies. Average frequencies are given when populations were sampled in multiple years. Numbers one to eight indicate populations where specimens were collected for quantification of physiological parameters (further details of these populations are provided in Table 1).

Frequencies and densities of males and female morphs were determined at 58 populations during the reproductive seasons of 2009 and 2010. This was done along a linear and continuous 1100 kilometre transect in the central to Eastern part of the species' distribution range, in Ontario and Quebec, Canada (Figure 1). Along this transect, annual mean temperatures range from 6.5°C at the south-west up to 0.8°C at the north-east. In addition, frequency and density data of 31 additional populations in Ontario, Quebec and New Brunswick that were sampled in 2004 and 2007 were used from Iserbyt *et al.* [38]. We aimed to sample populations minimally five kilometre separated from one another (mean ± SE: 13.0±1.1 km) and not being part of the same lake or river system. No specific permits were required for the described field studies. Neither were any of the locations privately owned or protected in any way.

### Population parameters

Frequencies and densities of males and female morphs were determined at each site as described by Van Gossum *et al.* [49]. Shortly, an insect net was swept transcribing ‘eight-shaped’ figures, while walking slowly through the shoreline vegetation and recording the time elapsed. Sex, morph and age class (immature or mature) of each individual *N. irene* netted was noted. We aimed to catch at least 30 females at each site (mean ± SE = 62±3). This allowed calculating six population parameters either based on matures or matures plus immature [38]: i.e. the proportion of females being andromorph, the ratio of andromorphs to males (i.e. mimics to models), the ratio of males to females (operational sex ratio, OSR), the population and male density (respectively number of individuals and number of males caught per time unit) and finally the proportion of mature individuals. Density and sex ratio are considered relevant proxies of male harassment, because when males are more numerous or when the number of males per female increases, females are expected to be approached more frequently by mate-searching males [62]–[64]. The proportion of mature individuals can be used as surrogate for moment in the reproductive season, with a proportion equal to zero at the onset and a proportion of one at the end of the season.

### Weather parameters

Given that female morph frequencies and physiological parameters (see further) can be influenced by long-term as well as short-term weather conditions [32], [20], [47], we obtained detailed weather data from the Canadian National Climate Data and Information Archive (http://www.climate.weatheroffice.ec.gc.ca). The average distance between our 89 study populations and the closest weather station was 16.8±1.0 km. Long-term annual and seasonal (winter, spring, summer and autumn) climate normals were derived from the weather stations closest to each site. These climate normals are the arithmetic averages of weather elements over the 30-year interval (1971–2000). Our extracted dataset includes annual and seasonal temperature (mean, minimum and maximum) and precipitation averaged over this thirty-year period. To obtain short-term weather data we derived seasonal mean precipitation and mean, minimum and maximum temperature, during the preceding year (starting with the summer of 2009). Additionally, on a very short-term time scale we derived daily precipitation, and mean, minimum and maximum temperatures for four periods: the day of capture, the day of capture plus the preceding day, the preceding two days and the preceding three days.

### Physiological parameters

To quantify physiological parameters, individuals were collected at a subset of eight sites along our transect (Table 1; Figure 1). We selected these populations based on the minimal distance between them (min: 65 km; mean ±1SE = 93±14 km), in such a way that large variation was present in both, weather and population parameters (Table 1). This large variation provides us with a good opportunity to explore morph-specific variation in the physiological estimates with respect to these ecological variables. The eight selected sites may thus be viewed as a representative subset of the total population dataset (N = 89) along our transect. Minimally 20 individuals (24.1±0.5) of each female morph were collected in 2010. These were all mature individuals judged by the brightness of their body colours and stiffness of the wings [57]. Mate status at the moment of capture, i.e. being single or mated, was noted for every female. Each female was stored separately and preserved immediately in liquid nitrogen in the field. Afterwards, all individuals were further stored at −80°C in the laboratory for further use.

**Table 1:** Ecological parameters of the eight populations where individuals were collected during the reproductive season of 2010 for quantification of physiological parameters.

Site name	Sample date	Afreq	Mdens	Temp	Prec
1. Otter Marsh	18–19/6	33.8	28.8	6.5	948
2. Alexandria	22-Jun	36.6	27.0	5.8	960
3. Quebec City	30/6 & 4/7	62.7	10.2	4.7	1170
4. Tourville	24-Jul	22.8	13.4	2.6	1219
5. St.-Hilarion	06-Jul	25.5	8.9	2.5	986
6. Rimouski	21–22/7	12.6	13.9	1.6	1165
7. Forestville	12-Jul	22.0	5.4	2.6	1084
8. Hauterive	15-Jul	51.7	11.9	1.5	1014

These parameters include andromorph frequencies (%) and male density (individuals/minute), based on summed immature and mature individuals, as well as annual temperature (°C) and precipitation (mm) normals. Site numbers correspond with the numbers in Figure 1.

We studied three key physiological parameters related to investments in immune function (phenoloxidase (PO) activity), in energy storage and fecundity (abdomen lipid and protein content) and in flight muscles (thorax protein content) that are strong candidates to affect fitness in damselflies [32], [54], [55], [65]. PO activity is one of the most important components of insect immune function [66]–[68]. Lipids are the most important form of energy storage in both the adult and the egg stage [69], [70]. Proteins are the major component of flight muscles [71] and are important for the development of the eggs [72]. Because flight muscles make up to 95% of the thorax mass [73], thorax protein content can be seen as a proxy for investment in flight muscles. Abdomen protein and lipid content reflect the investment in fecundity. All three physiological parameters have been shown to be influenced by male harassment [32], [74] and weather conditions [75], [76], and may potentially differ between morphs [29], [32,].

To quantify PO activity, we closely followed the protocol by Stoks *et al.* [77], here optimised for *N. irene*. Specifically, the thorax was homogenised using a hand-held pistil and 0.3 ml cacodylate buffer was added (0.01 mol/l Na-Coc, 0.005 mol/l CaCl_2_). The cell walls were removed via centrifugation (4°C, 13000 rpm, 10 min). Each well of a 96-well microtiterplate was filled with 100 µl sample supernatant, 35 µl PBS buffer, 5 µl chymotripsine (5 mg/ml) and after five minutes 60 µl L-Dopa (dihydrophenyl-l-alanine; 10 mM in cacodylate buffer). The reaction proceeded for 30 minutes at 30°C. Readings were taken every 10 seconds on a temperature-controlled microplate reader at 490 nm. Enzyme activity was measured as the slope during the linear phase of the reaction when the enzyme catalyses the transition from L-DOPA to dopachrome.

Protein content was quantified separately in thorax (mainly flight muscles) and abdomen (mainly eggs) using the Bradford method [78]. Therefore, 5 µl of the homogenised sample was added to 155 µl milli-Q-water and 40 µl Bradford reagent (Sigma®, San Louis – USA). The absorbance was read at 595 nm after 10 minutes on a microplate reader. Concentrations were calculated from standard curves of bovine serum albumine (United States Biochemical Corp, Bath – UK).

Abdomen lipid content was assayed using the protocol described in Bligh & Dyer [79]. 200 µl of the homogenised sample was mixed with 400 µl chloroform, 400 µl methanol and 200 µl milli-Q-water to dissolve the lipids. Lipids were precipitated via centrifugation (4°C, 13000 rpm, 5 min). 200 µl of the lower chloroform fraction was mixed with 500 µl concentrated H_2_SO_4_ and was incubated for 15 minutes at 200°C. Then 2 ml milli-Q-water was added and subsequently 200 µl of each sample was read at 405 nm on a microplate reader. Concentrations were calculated from standard curves of tripalmitine (Acros Organics, Geel– Belgium). Thorax lipids could not be quantified because all homogenised thorax sample was used to measure PO activity and protein content.

All physiological parameters were assayed twice per individual and the mean of both readings was used in all further analyses (all repeatabilities >0.83 [80]). A digital picture of the right hind wing was taken of each individual (Nikon D70/Tamron macro lens 90 mm 1∶2.8). Using ImageJ 1.38× [81], wing length was determined from the second antenodal cross vein to the stigma; for more detail see [61].

### Statistical analyses

We first tested for spatial autocorrelation to examine geographical dependency in the studied population and long-term weather parameters [82]. Therefore, we used the Morans' I index, with I-values significantly different from zero indicating that spatial heterogeneity in the ecological parameters increases with distance among populations. Nearby populations are thus expected to be more similar than populations further apart. Population parameters were averaged per site when sampled in multiple years and tested for spatial autocorrelation with a lag distance of 5 km. This value equals our minimal distance between nearby population and is much higher than the average dispersal distance of comparable zygopteran damselflies in a network of ponds [83]. In addition, we tested for clinal variation, i.e. latitudinal and longitudinal effects, in these ecological parameters. We accounted for sampling over multiple years by treating site as a random factor in the mixed models.

In a next step, we explored to what extent spatial variation in morph frequencies could be explained by linear and quadratic effects of latitude and longitude, harassment proxies (OSR and densities) and weather parameters (temperature and precipitation normals). As expected, several of these parameters were strongly correlated and may therefore not be included together in one regression model, because of multi-collinearity problems [84]. To make an a priori selection among the explanatory variables and meanwhile avoiding problems with multi-collinearity, we used two independent methods which have been proven to be successful in the past: classification and regression trees (CART) [85]–[87] and partial least squares regressions (PLS) [88], [89]. CART explains variation of a single response variable by repeatedly splitting the data with the best predictive variables into more homogeneous groups. PLS is a regression technique developed to deal with many explanatory variables and one or several response variables. Predictors selected by the PLS that are highly correlated (R^2^>0.9) are considered to have equal explanatory value. In such cases, the variable that best corresponded with the CART analyses was selected as best predictor. CART and PLS both selected the same best predictors in our dataset (see results). Therefore, these analyses can be seen as replicated and independent methods, which increases the robustness of our results. Subsequently, we tested whether the two best predictive parameters had a significant effect on the spatial variation in morph frequencies using separate general linear mixed models (GLM). These are basically multiple regression analyses, in which we controlled for possible within-season variation in morph frequencies [49], by including the proportion of mature individuals as linear and quadratic effects into the model. Also, given that some populations were sampled in multiple years, source population was treated as a random variable.

The ecological variables that could best explain variation in morph frequencies were also used to analyse variation in the physiological parameters. Therefore, we performed ANCOVA models, including morph, mate status and the selected ecological variables, plus interactions with morph. Source population was treated as a random variable. To correct for individual and morph-specific differences in size, protein and lipid contents were divided by wing length [61], [90]. To correct for individual and morph-specific differences in protein content when analyzing the enzyme PO, we calculated PO residuals obtained by regressing PO activity against thorax protein content. Correcting PO for wing length as the other physiological parameters did not change the outcome of the analyses. Similarly, using protein or lipid residuals obtained by the regression against wing length, did not alter the conclusions. All analyses were performed in SAS 9.2 (SAS Institute Inc, Carry, NC, USA), except for the CART analyses which were completed in SPSS 18.0 (SPSS Inc, Chicago, IL, USA).

## Results

### Variation in morph frequencies

Considerable spatial variation in the frequency of andromorph females was identified ranging from 0.0 to 62.7% (mean ± SE: 8.8±1.2%; see Figure 1). No sign of spatial autocorrelation in morph frequencies was detected, nor were there any effects of latitude or longitude (Table 2). Similar patterns were observed with the ratio of andromorphs to males and the OSR (Morans' I, latitude and longitude, all p>0.05). In contrast, male density and annual temperature normals both decreased towards Eastern and Northern directions, while annual precipitation normals increased towards the East. Strong signals of spatial autocorrelation were detected in these three ecological parameters (Table 2). Similar patterns were observed for population density and all seasonal mean, minimum and maximum temperature and precipitation normals (Morans' I, latitude and longitude, all p<0.0001).

**Table 2:** Outcome of the spatial autocorrelation analyses for andromorph frequency, male density, annual temperature and precipitation normals, using Morans' I index.

Parameter (α)	Morans' I	Latitude	Longitude
Afreq (p = 0.05)	I = 0.14, Z = 0.6, p = 0.54	F_1,40_ = 0.36, p = 0.55	F_1,40_ = 0.24, p = 0.63
Mdens (p = 0.02)	I = 0.88, Z = 3.4, p = **0.0007**	F_1,40_ = 26.5, p<**0.0001**	F_1,40_ = 13.4, p = **0.0007**
Temp (p = 0.01)	I = 0.97, Z = 3.7, p = **0.0002**	F_1,87_ = 372.1, p<**0.0001**	F_1,87_ = 42.6, p<**0.0001**
Prec (p = 0.03)	I = 0.71, Z = 2.8, p = **0.005**	F_1,87_ = 1.02, p = 0.32	F_1,87_ = 11.3, p = **0.001**

Latitudinal and longitudinal effects in these ecological parameters are tested with mixed models. Significant effects are indicated in bold. Given that we tested each parameter four times, we also provide between brackets the adjusted α threshold values based on the sequential Bonferroni correction [91]. Similar patterns were observed for other population and weather parameters (see results).

**Figure 2:**
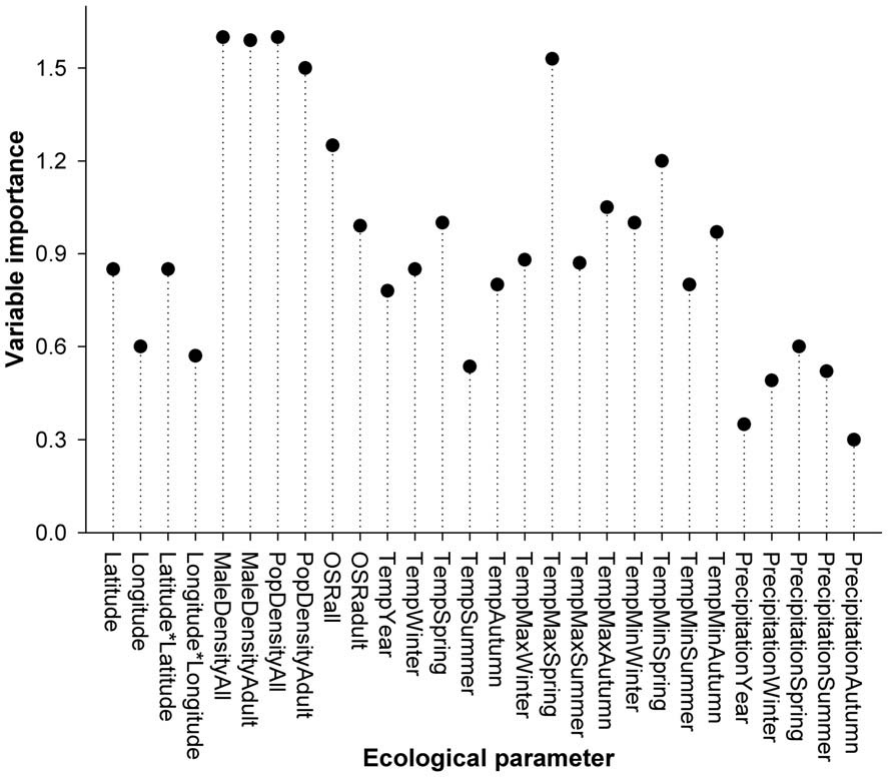
Variable importance plot of the partial least squares (PLS) regression explaining spatial variation in morph frequencies. Conform the CART analysis, mature male density and maximum spring temperature were selected as most important explanatory variables. Note that all density estimates are highly correlated (all R^2^>0.9). Hence, only adult male density is used in further analyses (see methods).

**Figure 3:**
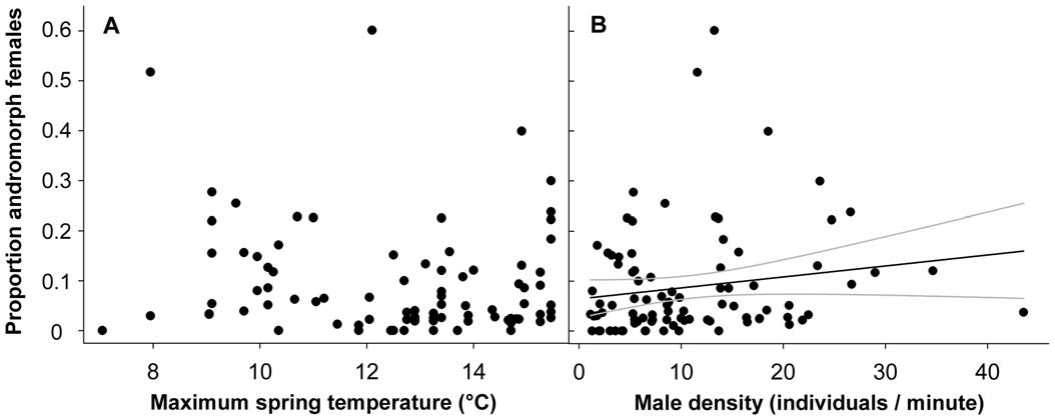
Spatial variation in andromorph frequencies plotted against maximum temperature normals in the spring season (A) and mature male density (B). These variables explained most of the spatial variation in morph frequencies, based on the CART and PLS analyses. Average population morph frequencies are presented when sampled over multiple years. Significant regression fit and 95% confidence interval is presented in panel (B).

The CART analysis put forward adult male density (split at 11.2 individuals/minute) as most important variable for explaining the spatial variation in morph frequencies, followed by maximum spring temperature normals (11.0°C). These results exactly correspond with the selected predictors of the PLS analysis (see Figure 2). Overall, precipitation regimes had very limited explanatory value (Figure 2). From the two selected ecological parameters, maximum spring temperature did explain only a minor, non-significant, part of the variation in andromorph frequencies (GLM: F_1,40_ = 2.38, p = 0.13; Pearson correlation: R^2^ = 0.02; Figure 3A). Andromorph frequencies increased with mature male densities (F_1,40_ = 5.55, p = 0.023; R^2^ = 0.04; Figure 3B). The proportion of mature individuals, as a controlling factor for seasonal variation, had no effect on morph frequencies (linear: F_1,39_ = 2.72, p = 0.11 and quadratic: F_1,38_ = 0.84, p = 0.36).

### Variation in physiological parameters

**Figure 4:**
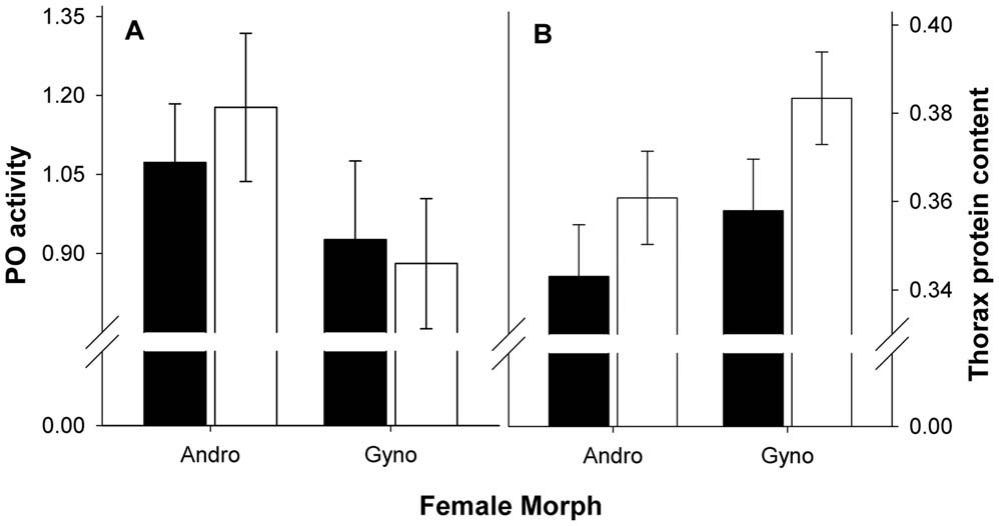
Differences between single (black) and mated (white) female morphs in PO activity (A) and thorax protein content (B). Values are based on the least squares means (±1SE) of the final general linear mixed models as presented in Table 2. For graphical clearness, mean values of PO activity (residuals obtained by regressing PO activity against thorax protein content) are summed by one.

Andromorphs had significantly, ca. 10% higher PO activity levels compared to gynomorphs (Table 3, Figure 4). The reverse pattern was found for thorax protein content, which was over 5% lower for andromorphs relative to gynomorphs. No morph-specific differences were found in abdominal physiological traits (Table 3). Although mature male density was the only significant ecological parameter that could explain part of the spatial variation in morph frequencies, this density effect did not differentially affect the physiological parameters of both female morphs (see interactions, Table 3). Neither did any of the various other studied population parameters, long-term and short-term weather parameters affect physiological parameters of both female morphs in contrasting ways (results not shown). Finally, mated females had significantly higher thorax protein content, compared to single females (Table 3, Figure 4B).

**Table 3:** Results of the general linear mixed models explaining variation in the four physiological parameters.

Effect	d.f.	F	p
PO activity			
Morph	364	5.52	**0.019** (0.02)
Status	362	0.07	0.79
Mdens	364	1.48	0.22
Thorax protein content			
Morph	382	6.82	**0.009** (0.01)
Status	382	7.94	**0.005** (0.01)
Mdens	382	0.61	0.44
Abdomen protein content			
Morph	389	0.32	0.57
Status	387	0.12	0.73
Mdens	390	0.69	0.41
Abdomen lipid content			
Morph	376	0.00	0.95
Status	377	2.81	0.09
Mdens	377	1.11	0.29

Explanatory variables include female morph, mate status, mature male density (Mdens) and both interactions with female morph. Morph-by-status and morph-by-density interactions were never significant (all P>0.1). The numerator degrees of freedom is one in all cases, d.f. in the table refers to the denominator degrees of freedom. Significant explanatory variables are indicated in bold. Given that we tested each effect for four response variables, we also provide between brackets the adjusted α threshold values based on the sequential Bonferroni correction [91].

## Discussion

Our results indicate large spatial variation in morph frequencies in a geographical rather unstructured way, which could be explained only weakly by numerous ecological parameters. These ecological parameters did not affect physiological fitness-related traits of both female morphs in contrasting ways. However, we showed a geographically consistent morph-specific trade-off between investment in immune function and in flight muscles. In what follows, we will discuss the above findings in a broader evolutionary context.

While most of the investigated ecological parameters showed spatial autocorrelation, this pattern was not present in female morph frequencies. Moreover, and contrary to our expectations, we found generally little proof for weather and population parameters being important in explaining biogeographic patterns in female morph frequencies. Our results may therefore indicate the importance of stochastic effects causing spatial frequency fluctuations. Alternatively, female morphs may be adapted to highly variable and currently unidentified small-scale environmental conditions. The latter alternative explanation may seem less likely as we included most of the known weather and population parameters likely to differentially affect the colour morphs, although we acknowledge that some factors may be overlooked. Considerable variation in morph frequencies in a spatially structured manner has been reported in taxonomically diverse systems, such as birds [19], mammals [21], insects [18], [39], molluscs [13] and plants [92]. Such studies provide preliminary support for selection underlying these polymorphisms. The clinal variation in morph frequencies in female damselflies has been related with variation in ambient temperature in *Ischnura elegans* [47] and altitude related solar radiation in *Megalagrion calliphya* [20]. In another approach to understand these biogeographical patterns, population genetic studies compared genetic variability of neutral loci with morph frequencies at local spatial and temporal scales [93]–[96]. While direction, form and magnitude of selection differed among studies, they jointly argued against drift to maintain multiple female morphs in natural populations. Specifically for *N. irene*, Wong et al. [50] suggested that on a local scale spatially variable selection operates on different morphs, perhaps mediated by adaptation to variable local environmental conditions, frequency- and density-dependent selection regimes or a combination of those. A similar conclusion could be drawn for *I. senegalensis* [39] and *I. elegans* at a large spatial scale [7], in which the strength of divergent selection differed among regions. Similar to our observations, one of the female morphs is extremely rare or even absent in some parts of *I. elegans'* distribution area [7], [15]. This could either be caused by local selection acting against this rare morph, or by stochastic effects during colonization of certain areas; for lizards see also [97]. In accordance, a recent population genetic study indicated that the extreme continental frequency variation (from 0% to ∼100%) in *N. irene* could in part be explained by such stochastic effects [45]. The lack of spatial autocorrelation in morph frequencies and the weak explanatory value of ecological parameters in the current study strengthen this idea.

Nonetheless, and in accordance with a number of studies, we found, admittedly weak (R^2^ = 0.04; see figure 3B), but significant co-variation between spatial variation in andromorph frequencies and male density [38], [49], [59], [93], [98]. Evidently, this relationship does not prove causality. However, theoretical and empirical studies support that the intensity of sexual conflict rises with male density, thereby reducing female fitness components [32], [62]–[64]. Harassment selection may thus be too low or even absent in populations with low male density [99]. Drift may get the upper hand in such conditions, generating fixation of a given morph [2], [14]. This may also explain why 13 out of 89 populations are monomorphic in the current study.

To point at selection maintaining spatial variation in the polymorphism, relative fitness components of the morphs should also differ in relation to the supposed selective agent [25], [32], [51]. However, in the present study no morph-specific influences of male density (or other population parameters, nor short- and long-term weather parameters) on physiological traits were detected. Contrarily, body condition measures in the damselfly *Enallagma cyathigerum* were differentially affected by short-term ambient temperature in both morphs [32]. One reason for this difference between studies may be the smaller variation in weather parameters in the current study (e.g. 11–22°C in [32], vs 17–23°C in the current study). However, in line with the current results, neither did other recent studies find such morph-specific influences of environmental conditions on several behavioural and life-history traits [48], [100], [101].

With regard to trade-offs between fitness-related physiological traits contributing to maintaining the polymorphism at a local scale, our physiological data suggest a morph-specific and geographically consistent trade-off where andromorphs, relative to gynomorphs, tend to invest more in immune function and less in flight muscles. Because traits related to immune function and flight muscles are costly to produce and maintain [102]–[104], our results likely reflect an energy-allocation trade-off [105], [106]. Interestingly, similar trade-offs have been suggested in polymorphic butterflies [107], but see [108] and are particularly well described in several species of polymorphic lizards. In these latter model species, colour morphs may differentially allocate resources towards traits related to immune function versus life-history, morphology or performance-related traits (*Uta stansburiana*: [27]; *Podarcis muralis*: [30]; *Anolis sagrei*: [29]). These lizard studies suggest that correlational selection driven by density of neighbouring individuals favours successful and alternative trait combinations among morphs [27], [29]. In our study, both morphs may differentially allocate resources to traits related to immune function and flight muscles because of differences in degree of male sexual harassment and/or differences in behavioural strategies to avoid this cost. In this scenario, gynomorphs would invest more in flight muscles that enhance aerial competitive ability [71], [73], [109] to fend of harassing males. This effect was even stronger for mated females, perhaps because males particularly search and mate females that are most active and in best condition [110]. In line with the suggested enhanced flight ability, *N. irene* gynomorphs display more refusal behaviour when they are the majority morph [111], see also [100]. Also in *I. elegans*, female morphs differ in behavioural tactics in order to escape from excessive male harassment [110], [112]. Specifically andromorphs occupy less open habitat, fly within shorter ranges and directly fend off approaching males [112]. These observed behavioural differences may relate to differential investment in flight muscles and differential exposure to parasites [113] and thus investment in immune function.

Whatever the underlying mechanism, our data and other studies suggest correlational selection favouring successful and alternative trait combinations among female morphs [114]. This multivariate suit of traits involve physiological (this study) as well as behavioural [111], morphological [60], [61] and life-history [115] traits. Identifying trade-offs among fitness-related traits may increase our knowledge how polymorphisms are maintained, because they may generate morph-specific fitness optima in phenotypic space [27], [29], [30].

### Conclusions

Despite many studies, the mechanisms underlying female polymorphism in damselflies are still under debate and this study area urgently needs to open its perspective and accept multiple non-exclusive explanations [7], [20], [35], [36]. In the present study, we provided several new insights. We first showed that morph frequencies are spatially unstructured and are only weakly related to numerous investigated ecological parameters. This may indicate that morph frequencies either vary randomly caused by stochastic processes or alternatively, that female morphs are adapted to local ecological conditions that largely vary at a small spatial scale. Furthermore we showed that ecological parameters did not differentially affect fitness-related physiological traits of both female morphs. Instead, we documented geographically consistent morph differences in physiology reflecting a trade-off between investments in immune function and in flight muscles. Our results share much resemblance with results on polymorphic lizards and highlight an overlooked candidate mechanism contributing to the maintenance of multiple female morphs within damselfly species.
